# Synthesis of δ-Oxo-1,1-bis(triflyl)alkanes and Their Acidities

**DOI:** 10.3390/molecules181215531

**Published:** 2013-12-13

**Authors:** Hikaru Yanai, Masaya Fujita, Arata Takahashi, Min Zhang, Masaaki Mishima, Akira Kotani, Takashi Matsumoto, Takeo Taguchi

**Affiliations:** 1 School of Pharmacy, Tokyo University of Pharmacy and Life Sciences, 1432-1 Horinouchi, Hachioji, Tokyo 192-0392, Japan; 2 Institute for Materials Chemistry and Engineering, Kyushu University, 6-10-1 Hakozaki, Higashi-ku, Fukuoka 812-8581, Japan

**Keywords:** carbon acid, triflyl group, gas phase acidity, p*K*_a_

## Abstract

The reaction of 1,1-bis(triflyl)ethylene generated *in situ* with enolizable carbonyls yielded δ-oxo-1,1-bis(triflyl)alkane derivatives. Their acidities in both the gas and solution phases were determined.

## 1. Introduction

**Scheme 1:**
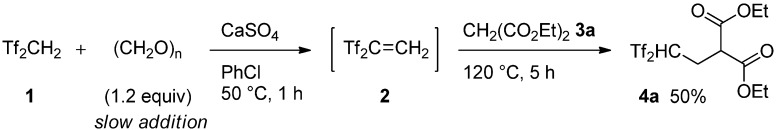
Koshar’s synthesis of bis(triflyl)ethylated malonate **4a**.

The bis(triflyl)methyl (Tf_2_CH; Tf = CF_3_SO_2_) group is known to be a strong C–H acidic functionality due to the *gem*-disubstitution of a carbon atom by two triflyl groups. This type of carbon acid (C–H acid) shows notably strong acidity not only in the gas-phase [1,2] but also in solution-phase [3]. For example, the gas-phase acidity Δ*G*_acid_ of Tf_2_CH_2_ (**1**) has been determined to be 300.6 kcal mol^−1^. Compared to the value of sulfuric acid (302.2 kcal mol^−1^), this somewhat lower value means that Tf_2_CH_2_ **1** performs as a superacid in the gas-phase. The p*K*_a_ of **1** in DMSO is also measured as 2.1 and it works as a better proton donor relative to trifluoroacetic acid (p*K*_a_ in DMSO = 3.45). On the basis of this feature, some powerful Brønsted acid catalysts containing Tf_2_CH functionalities such as Tf_2_CHC_6_F_5_ [4,5,6], Tf_2_CHCH_2_CHTf_2_ [7,8,9,10], and multiple carbon acids [11,12,13] were developed. Compared to the corresponding nitrogen acid Tf_2_NH and oxygen acid TfOH, these carbon acids show excellent catalyst performance in several synthetic reactions, including the Mukaiyama aldol reaction, the Friedel–Crafts acylation, and esterification. However, the synthesis and purification of such strongly acidic carbon acids are not so easy [14]. For example, Koshar and co-workers reported that *in situ*-formation of 1,1-bis(triflyl)ethylene (**2**) by the reaction of Tf_2_CH_2_ (**1**) with paraformaldehyde in the presence of CaSO_4_ and the subsequent one-pot reaction with diethyl malonate (**3a**) gave the bis(triflyl)ethylated malonate **4a** in poor yield (Scheme 1) [15].

**Figure 1:**
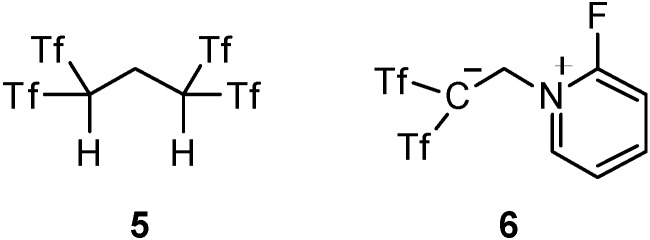
Structures of 1,1-bis(triflyl)ethylating reagents.

Since this reaction required harsh conditions for the effective generation of the alkene intermediate **2**, the yield of **4a** was not very high. To overcome this problem, we reported that 1,1,3,3-tetrakis(triflyl)propane (**5**, Figure 1) [16,17] can be used as not only an acid catalyst, but also a very effective reagent for *in situ*-generation of Tf_2_C=CH_2_ (**2**) via a retro-Michael type reaction. Recently, zwitterion **6** (Figure 1) was also developed for the same use [18]. These reagents can be easily prepared on multi-gram scale from commercially available Tf_2_CH_2_ (**1**) in one step.

Herein we report an improved synthesis of δ-oxo-1,1-bis(triflyl)alkanes via bis(triflyl)ethylation reaction of enolizable carbonyls with tetrasulfone **5**. Furthermore, both gas-phase acidity and p*K*_a_ values in a DMSO solution of some of the prepared carbon acids were determined.

## 2. Results and Discussion

### 2.1. Improved Synthesis of δ-Oxo-1,1-bis(triflyl)alkanes

**Scheme 2:**
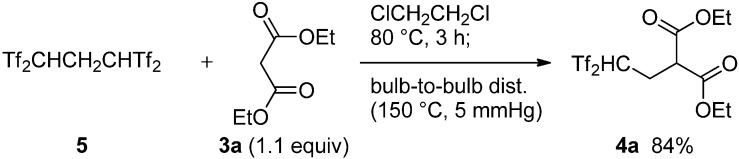
Improved synthesis of bis(triflyl)ethylated malonate **4a**.

Keeping Koshar’s original work in mind, we first examined the 2,2-bis(triflyl)ethylation reaction of diethyl malonate (**3a**, Scheme 2). Notably, the reaction of **3a** with Tf_2_CHCH_2_CHTf_2_ (**5**) was smoothly completed within 3 h at 80 °C. In this case, we observed complete consumption of tetrasulfone **5** and quantitative formation of Tf_2_CH_2_ (**1**) and the desired carbon acid **4a** by ^19^F-NMR analysis of the crude mixture. This mixture was successfully purified by bulb-to-bulb distillation (150 °C at 5 mmHg) using a Kugelrohr oven to give acceptably pure carbon acid **4a** in 84% yield. Compared to Koshar’s procedure, the use of tetrasulfone **5** instead of Tf_2_CH_2_/paraformaldehyde resulted in a better yield of **4a** (50% *vs.* 84%) within a shorter reaction time.

**Figure 2:**
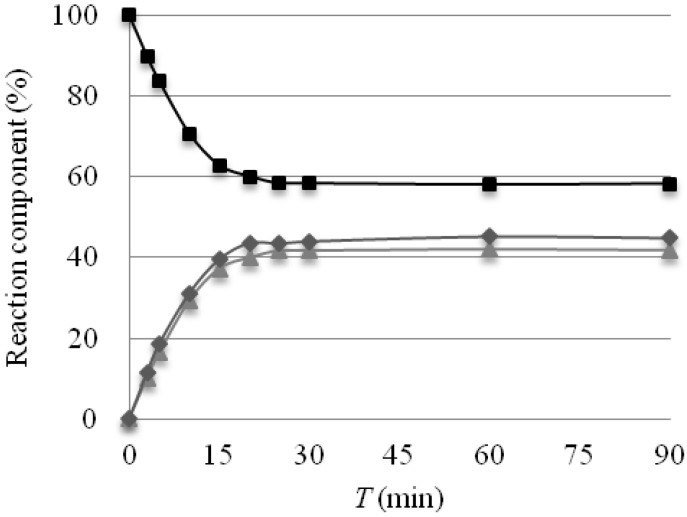
Reaction profile in a 0.01 M solution of Tf_2_CHCH_2_CHTf_2_ (**5**) in CDCl_3_ at 40 °C (squares, Tf_2_CHCH_2_CHTf_2_ (**5**); diamonds, Tf_2_CH_2_ (**1**); triangles, Tf_2_C=CH_2_ (**2**)).

Smooth formation of the carbon acid **4a** in the present case could be attributed to rapid formation of alkene intermediate **2** in solution phase from tetrasulfone **5**. For instance, ^1^H-NMR analysis of a solution of **5** in CDCl_3_ at 40 °C revealed very rapid formation of **2** in a reversible manner (Figure 2). When this mixture was left for 20 min at 40 °C, tetrasulfone **5** partly decomposed to Tf_2_CH_2_ (**1**) and alkene **2** to give an equilibrium mixture between **5** and **1**/**2** without formation of any side products and its equilibrium constant *K*_eq_ was calculated as 3.21 × 10^−3^.

Since the product yield in the bis(triflyl)ethylation with Tf_2_CHCH_2_CHTf_2_ (**5**) was better than that in Koshar’s original procedure using Tf_2_CH_2_ (**1**) and paraformaldehyde, we carried out the reaction of several active methylenes with tetrasulfone **5**. Selected results are summarized in Table 1. When some dialkyl malonates such as **3b** and **3c** were treated with tetrasulfone 5 at 80 °C in 1,2-dichloroethane, the desired bis(triflyl)ethylated products **4b** and **4c** were obtained in 84% and 98% yield, respectively (entries 1 and 2). In the case of dimethyl malonate (**3b**), the product **4b** was isolated after standard bulb-to-bulb distillation (method A). Although distillation of dibenzyl derivative **4c** was problematic due to its high boiling point, acceptably pure **4c** was obtained by removal of Tf_2_CH_2_ (**1**) and remaining **3c** by a Kugelrohr oven (120 °C at 2 mmHg; method B). Under similar conditions, carbon acid **4d** derived from phosphonyl acetate **3d** was isolated in 57% yield using method A (entry 3). The reactions of β-ketoesters and of 1,3-diketones proceeded under more mild conditions in CH_2_Cl_2_. For example, the products **4e**–**g** derived from β-ketoesters were obtained in excellent yields by the reaction at 40 °C (entries 4–6). Likewise, the reactions of 1,3-diketones **3h** and **3i** with tetrasulfone **5** completed at room temperature to give the corresponding products **4h** and **4i** in 74% and 80% yields, respectively (entries 7 and 8).

**Table 1:** Reaction of Tf_2_CHCH_2_CHTf_2_ **5** with 1,3-dicarbonyl compound **3**.

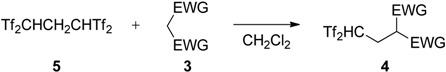							
Entry	3		Temp. (°C)	Time (h)	Method *^a^*	4	Yield *^b^* (%)
1 *^c^*	**3b**	CH_2_(CO_2_CH_3_)_2_	80	8	A	**4b**	84
2 *^c^*	**3c**	CH_2_(CO_2_Bn)_2_	80	8	B	**4c**	98
3 *^c^*	**3d**	CH_2_(CO_2_CH_3_)P(O)(OCH_3_)_2_	80	5.5	A	**4d**	57
4	**3e**	CH_2_(CO*t*-Bu)CO_2_CH_3_	40	2	A	**4e**	93
5	**3f**	CH_2_(CO*t*-Bu)CO_2_Et	40	5	A	**4f**	82
6	**3g**	CH_2_(COPh)CO_2_Et	40	4.5	B	**4g**	86
7	**3h**	CH_2_(CO*t*-Bu)_2_	Rt	4	A	**4h**	73
8	**3i**	CH_2_(CO*i*-Pr)_2_	Rt	2.5	A	**4i**	80

*^a^* Method A; Product was isolated by bulb-to-bulb distillation. Method B; Product was purified by distillative removal of Tf_2_CH_2_ **1** and remaining **3** using a Kugelrohr oven; *^b^* Isolated yield; *^c^* Reaction was carried out in 1,2-dichloroethane.

**Scheme 3:**
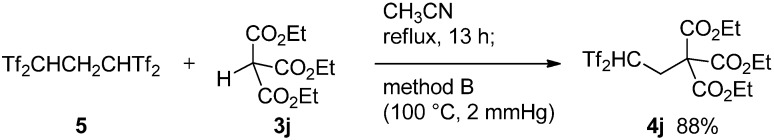
Bis(triflyl)ethylation of triester **3j**.

As shown in Scheme 3, it should be noted that less reactive triester **3j** smoothly converted to the corresponding bis(triflyl)ethylated product **4j** in 78% yield under the present conditions. In this case, we found that the use of acetonitrile instead of 1,2-dichloroethane gives a better yield of **4j**.

### 2.2. Gas-Phase and Solution-Phase Acidities of Carbon Acids

The gas-phase acidity Δ*G*_acid_ established with the use of the FT-ICR technique [1,2] is known as an extensive scale for strong acids. We measured Δ*G*_acid_ values of some select carbon acids (Table 2). The values of triflylated methanes were reduced by increasing in the number of triflyl group (TfCH_3_, 339.8 kcal mol^−1^ [19]; Tf_2_CH_2_ (**1**), 300.6 kcal mol^−1^ [1]; Tf_3_CH, 289.0 kcal mol^−1^ [2]) (entries 1, 2, and 7). The value of Tf_2_CHCH_2_CHTf_2_ (**5**) was recently revised to 290.2 kcal mol^−1^ and its acidity is notably stronger than that of Tf_2_CH_2_ **1** [20]. On the other hand, δ-oxo-1,1-bis(triflyl)alkanes **4b**, **4e**, and **4h** showed very similar acidities in gas-phase compared to Tf_2_CH_2_ (**1**). That is, established Δ*G*_acid_ values of these compounds were 299.6 kcal mol^−1^, 300.3 kcal mol^−1^, and 300.4 kcal mol^−1^, respectively (entries 3–5). This finding suggests that the difference of carbonyl functionalities in the structures of δ-oxo-1,1-bis(triflyl)alkanes is not critical factor for their gas-phase acidities. Therefore, the symmetrical structure of **5** plays an important role for its significantly enhanced acidity (the statistical effect). In addition, the p*K*_a_ value of carbon acid **4b** in DMSO solution was determined as 2.16 by the voltammetric method [21,22]. This also means that the **4** is comparable in the acidity to **1** (p*K*_a_ = 2.1) in DMSO solution.

**Table 2:** The gas-phase acidities of carbon acids.

Entry	Carbon Acid	Δ*G*_acid_ (kcal mol^−1^)
1 *^a^*	TfCH_3_	339.8
2 *^b^*	Tf_2_CH_2_ (**1**)	300.6
3	Tf_2_CHCH_2_CH(CO*t*-Bu)_2_ (**4h**)	300.4
4	Tf_2_CHCH_2_CH(CO*t*-Bu)CO_2_CH_3_ (**4e**)	300.3
5	Tf_2_CHCH_2_CH(CO_2_CH_3_)_2_ (**4b**)	299.6
6 *^c^*	Tf_2_CHCH_2_CHTf_2_ (**5**)	290.2
7 *^d^*	Tf_3_CH	289.0

*^a^* Ref. [19]. *^b^* Ref. [1]. *^c^* Ref. [20]. *^d^* Ref. [2].

## 3. Experimental

### 3.1. General

All reactions were carried out under Ar atmosphere. Melting points were uncorrected. ^1^H- (400 MHz) and ^13^C-NMR (100 MHz) spectra were taken on a Bruker DPX 400 spectrometer, and chemical shifts were reported in parts per million (ppm) using CHCl_3_ (7.26 ppm) in CDCl_3_ for ^1^H-NMR, and CDCl_3_ (77.01 ppm) for ^13^C-NMR as an internal standard, respectively. ^19^F-NMR spectra were taken on a Varian Mercury 300 spectrometer (282 MHz for ^19^F), and chemical shifts were reported in parts per million using trifluoromethylbenzene (0 ppm) as a standard. Mass spectra were recorded by an electrospray ionization-time of flight (ESI-TOF) mass spectrometer (Micromass LCT). IR spectra were recorded by a JASCO FT/IR 4100 spectrometer. Column chromatography was performed on neutral silica gel (75–150 μm). Tf_2_CHCH_2_CHTf_2_ (**5**) was prepared from Tf_2_CH_2_ (**1**) by the reported procedure [15].

### 3.2. General Procedure for Bis(triflyl)ethylation Reaction of Enolizable Carbonyls

To a solution of carbonyl compound (1.0–2.0 equiv) in CH_2_Cl_2_ or 1,2-dichloroethane, Tf_2_CHCH_2_CHTf_2_ (**5**, 0.50 mmol) was added at room temperature. After stirring at room temperature to 80 °C, the reaction mixture was concentrated under reduced pressure. The resultant residue was purified by bulb-to-bulb distillation using a Kugelrohr oven to give bis(triflyl)ethylated product **4**.

*Diethyl 2-(2,2-bis(trifluoromethylsulfonyl)ethyl)malonate* (**4a**). According to the general procedure, this compound was obtained in 84% yield (190 mg, 0.420 mmol) by the reaction of Tf_2_CHCH_2_CHTf_2_ (**5**, 286 mg, 0.500 mmol) with diethyl malonate (83.5 μL, 0.55 mmol) in CH_2_Cl_2_ (0.10 mL) at 80 °C for 3 h and the following bulb-to-bulb distillation (140–160 °C at 5 mmHg). Colorless oil; IR (neat) *ν* 2989, 2944, 1746, 1397, 1214, 1115, 1024 cm^−1^; ^1^H-NMR (CDCl_3_) *δ* 1.29 (6H, t, *J* = 7.1 Hz), 2.91–2.98 (2H, m), 3.93 (1H, t, *J* = 7.8 Hz), 4.19–4.33 (4H, m), 5.73 (1H, t, *J* = 6.6 Hz); ^13^C-NMR (CDCl_3_) *δ* 13.9, 24.6, 47.5, 62.7, 74.4, 119.2 (q, *J*_C-F_ = 329.6 Hz), 167.5; ^19^F-NMR (CDCl_3_) *δ*−10.1 (6F, s); MS (ESI-TOF) *m*/*z* 453 [M+H]^+^; HRMS calcd. for C_11_H_15_F_6_O_8_S_2_ [M+H]^+^, 453.0113; found, 453.0054. Anal. calcd. for C_11_H_14_F_6_O_8_S_2_: C, 29.21; H, 3.12. Found: C, 29.24; H, 3.19.

*Dimethyl 2-(2,2-bis(trifluoromethylsulfonyl)ethyl)malonate* (**4b**). According to the general procedure, this compound was obtained in 84% yield (178 mg, 0.420 mmol) by the reaction of Tf_2_CHCH_2_CHTf_2_ (**5**, 286 mg, 0.500 mmol) with dimethyl malonate (62.9 μL, 0.55 mmol) in CH_2_Cl_2_ (0.20 mL) at 80 °C for 8 h and the following bulb-to-bulb distillation (160–170 °C at 5 mmHg). Colorless crystals (Et_2_O-hexane); Mp. 44.2–46.8 °C; IR (neat) *ν* 3007, 2962, 2854, 1747, 1440, 1395, 1215, 1114, 1036, 1004, 705 cm^−1^; ^1^H-NMR (CDCl_3_) *δ* 2.92–2.98 (2H, m), 3.81 (6H, s), 3.99 (1H, t, *J* = 7.8 Hz), 5.69 (1H, t, *J* = 6.6 Hz); ^13^C-NMR (CDCl_3_) *δ* 24.7, 47.3, 53.5, 74.3, 119.2 (q, *J*_C-F_ = 329.8 Hz), 167.8; ^19^F-NMR (CDCl_3_) *δ* −10.0 (6F, s); MS (ESI-TOF) *m*/*z* 425 [M+H]^+^; HRMS calcd for C_9_H_11_F_6_O_8_S_2_ [M+H]^+^, 424.9800; found, 424.9825. Anal. calcd. for C_9_H_10_F_6_O_8_S_2_: C, 25.48; H, 2.38. Found: C, 25.82; H, 2.57.

*Dibenzyl 2-(2,2-bis(trifluoromethylsulfonyl)ethyl)malonate* (**4c**). According to the general procedure, this compound was obtained in 98% yield (56.7 mg, 984 μmol) by the reaction of dibenzyl malonate (28.4 mg, 0.10 mmol) with Tf_2_CHCH_2_CHTf_2_ (**5**, 64.0 mg, 0.112 mmol) in CH_2_Cl_2_ (0.15 mL) at 80 °C for 8 h and the following removal of Tf_2_CH_2_ using a Kugelrohr oven (140–150 °C at 5 mmHg). Colorless oil; IR (neat) *ν* 3068, 3036, 2954, 1747, 1498, 1456, 1396, 1302, 1215, 1114, 750, 697 cm^−1^; ^1^H-NMR (CDCl_3_) *δ* 2.98 (2H, t, *J* = 7.2 Hz), 4.05 (1H, t, *J* = 7.2 Hz), 5.15 (2H, d, *J* = 12.3 Hz), 5.18 (2H, d, *J* = 12.3 Hz), 5.66 (1H, t, *J* = 7.2 Hz), 7.24–7.27 (4H, m), 7.30–7.34 (6H, m); ^13^C-NMR (CDCl_3_) *δ* 24.5, 47.6, 68.3, 74.3, 119.2 (q, *J*_C-F_ = 329.7 Hz), 128.4, 128.7, 128.8, 134.3, 167.2; ^19^F-NMR (CDCl_3_) *δ* −10.0 (6F, s); MS (ESI-TOF) *m*/*z* 599 [M+Na]^+^; HRMS calcd. for C_21_H_18_F_6_NaO_8_S_2_ [M+Na]^+^, 599.0245; found, 599.0247. Anal. calcd. for C_21_H_18_F_6_O_8_S_2_: C, 43.75; H, 3.15. Found: C, 44.09; H, 3.35.

*Methyl 2-(dimethoxyphosphoryl)-4,4-bis(trifluoromethylsulfonyl)butanoate* (**4d**). According to the general procedure, this compound was obtained in 57% yield (68.2 mg, 0.144 mmol) by the reaction of Tf_2_CHCH_2_CHTf_2_ (**5**, 145 mg, 0.253 mmol) with methyl 2-(dimethoxyphosphoryl)acetate (47.2 mg, 0.259 mmol) in 1,2-dichloroethane (0.40 mL) at 80 °C for 5.5 h and the following bulb-to-bulb distillation (190–210 °C at 5 mmHg). Colorless crystal (Et_2_O); Mp. 80.5–83.2 °C; IR (neat) *ν* 2964, 1741, 1394, 1342, 1216, 1115, 1053 cm^−1^; ^1^H-NMR (CDCl_3_) *δ* 2.86–3.04 (2H, m), 3.63 (1H, dt, *J*_H-P_ = 23.8 Hz, *J*_H-H_ = 7.8 Hz), 3.818 (3H, s), 3.821 (3H, d, *J*_H-P_ = 10.9 Hz), 3.85 (3H, d, *J*_H-P_ = 10.5 Hz), 6.21 (1H, t, *J* = 6.4 Hz); ^13^C-NMR (CDCl_3_) *δ* 23.2, 40.1 (d, *J*_C-P_ = 130.5 Hz), 53.5, 53.9 (d, *J*_C-P_ = 7.1 Hz), 54.0 (d, *J*_C-P_ = 6.4 Hz), 74.5, 119.2 (d, *J*_C-F_ = 329.8 Hz), 167.3 (d, *J*_C-P_ = 6.9 Hz); ^19^F-NMR (CDCl_3_) *δ* −10.3 (3F, s), −10.0 (3F, s); MS (ESI-TOF) *m*/*z* 475 [M+H]^+^; HRMS calcd. for C_9_H_14_F_6_O_9_PS_2_ [M+H]^+^, 474.9721; found, 474.9714. Anal. calcd. for C_9_H_13_F_6_O_9_PS_2_: C, 22.79; H, 2.76. Found: C, 22.63; H, 3.04.

*Methyl 2-(2,2-bis(trifluoromethylsulfonyl)ethyl)-4,4-dimethyl-3-oxopentanoate* (**4e**). According to the general procedure, this compound was obtained in 93% yield (73.4 mg, 0.163 mmol) by the reaction of Tf_2_CHCH_2_CHTf_2_ (**5**, 100 mg, 0.175 mmol) with methyl 4,4-dimethyl-3-oxopentanoate (31 μL, 0.19 mmol) in CH_2_Cl_2_ (0.15 mL) at 40 °C for 2 h and the following bulb-to-bulb distillation (150–160 °C at 5 mmHg). Colorless crystals (Et_2_O-hexane); Mp. 55.1–55.9 °C; IR (KBr) *ν* 2975, 2911, 2880, 1742, 1712, 1481, 1439, 1396, 1350, 1214, 1115, 970, 699, 676 cm^−1^; ^1^H-NMR (CDCl_3_) *δ* 1.21 (9H, s), 2.60 (1H, ddd, *J* = 15.0, 9.6, 4.5 Hz), 2.95 (1H, ddd, *J* = 15.0, 10.5, 3.6 Hz), 3.74 (3H, s), 4.53 (1H, dd, *J* = 10.5, 4.5 Hz), 5.68 (1H, dd, *J* = 9.6, 3.6 Hz); ^13^C-NMR (CDCl_3_) *δ* 25.8, 25.9, 45.8, 47.3, 53.2, 74.6, 119.20 (q, *J*_C-F_ = 329.8 Hz), 119.23 (q, *J*_C-F_ = 329.7 Hz), 169.2, 208.6; ^19^F-NMR (CDCl_3_) *δ* −10.2 (3F, s), −10.1 (3F, s); MS (ESI-TOF) *m*/*z* 451 [M+H]^+^; HRMS calcd. for C_12_H_17_F_6_O_7_S_2_ [M+H]^+^, 451.0320; found, 451.0320. Anal. calcd. for C_12_H_16_F_6_O_7_S_2_: C, 32.00; H, 3.58. Found: C, 31.63; H, 3.63.

*Ethyl 2-(2,2-bis(trifluoromethylsulfonyl)ethyl)-4,4-dimethyl-3-oxopentanoate* (**4f**). According to the general procedure, this compound was obtained in 82% yield (223 mg, 0.480 mmol) by the reaction of Tf_2_CHCH_2_CHTf_2_ (**5**, 336 mg, 0.587 mmol) with ethyl 4,4-dimethyl-3-oxopentanoate (104 μL, 0.59 mmol) in 1,2-dichloroethane (0.50 mL) at 40 °C for 5 h and the following bulb-to-bulb distillation (160–170 °C at 5 mmHg). Colorless crystals (Et_2_O-hexane); Mp. 34.7–36.5 °C; IR (KBr) *ν* 2979, 2942, 2911, 2876, 1738, 1712, 1480, 1397, 1350, 1213, 1114, 847, 781, 678 cm^−1^; ^1^H-NMR (CDCl_3_) *δ* 1.20 (9H, s), 1.24 (3H, t, *J* = 7.1 Hz), 2.58 (1H, ddd, *J* = 15.1, 10.3, 4.4 Hz), 2.95 (1H, ddd, *J* = 15.1, 11.2, 3.5 Hz), 4.18 (2H, q, *J* = 7.1 Hz), 4.50 (1H, dd, *J* = 11.2, 4.4 Hz), 5.71 (1H, dd, *J* = 10.3, 3.5 Hz); ^13^C-NMR (CDCl_3_) *δ* 13.7, 25.7, 25.9, 45.7, 47.4, 62.5, 74.6, 119.20 (q, *J*_C-F_ = 329.6 Hz), 119.24 (q, *J*_C-F_ = 329.8 Hz), 168.7, 208.7; ^19^F-NMR (CDCl_3_) *δ* –10.3 (3F, s), –10.0 (3F, s); MS (ESI-TOF) *m*/*z* 465 [M+H]^+^; HRMS calcd. for C_13_H_19_F_6_O_7_S_2_ [M+H]^+^, 465.0476; found, 465.0496. Anal. calcd. for C_13_H_18_F_6_O_7_S_2_: C, 33.63; H, 3.91. Found: C, 33.47; H, 4.17.

*Ethyl 2-benzoyl-4,4-bis(trifluoromethylsulfonyl)butanoate* (**4g**). According to the general procedure, this compound was obtained in 86% yield (86.1 mg, 0.178 mmol) by the reaction of Tf_2_CHCH_2_CHTf_2_ (**5**, 119 mg, 0.208 mmol) with ethyl 3-oxo-3-phenylpropanoate (44.0 mg, 0.229 mmol) in CH_2_Cl_2_ (0.15 mL) at 40 °C for 4.5 h and the following removal of Tf_2_CH_2_ (1) using a Kugelrohr oven (120 °C at 5 mmHg). Colorless oil; IR (neat) *ν* 3068, 2990, 2932, 1738, 1688, 1598, 1449, 1396, 1293, 1215, 1114, 1028, 774, 689 cm^−1^; ^1^H-NMR (CDCl_3_) *δ* 1.14 (3H, t, *J* = 7.1 Hz), 2.95–3.08 (1H, m), 3.08–3.16 (1H, ddd, *J* = 15.8, 8.8, 5.7 Hz), 4.16 (2H, q, *J* = 7.1 Hz), 5.02 (1H, dd, *J* = 8.8, 6.4 Hz), 5.62 (1H, dd, *J* = 8.0, 5.7 Hz), 7.53 (2H, t, *J* = 7.0 Hz), 7.64–7.68 (1H, m), 8.00 (2H, d, *J* = 7.0 Hz); ^13^C-NMR (CDCl_3_) *δ* 13.7, 24.7, 49.3, 62.7, 74.8, 119.22 (q, *J*_C-F_ = 329.8 Hz), 119.25 (q, *J*_C-F_ = 329.8 Hz), 128.9, 129.0, 134.5, 135.1, 168.1, 193.0; ^19^F-NMR (CDCl_3_) *δ* −10.1 (3F, s), −9.9 (3F, s); MS (ESI-TOF) *m*/*z* 485 [M+H]^+^; HRMS calcd. for C_15_H_15_F_6_O_7_S_2_ [M+H]^+^, 485.0163; found, 485.0156.

*4-(2,2-Bis(trifluoromethylsulfonyl)ethyl)-2,2,6,6-tetramethylheptane-3,5-dione* (**4h**). According to the general procedure, this compound was obtained in 73% yield (72.8 mg, 0.153 mmol) by the reaction of Tf_2_CHCH_2_CHTf_2_ (**5**, 119 mg, 0.208 mmol) with 2,2,6,6-tetramethylheptane-3,5-dione (48 μL, 0.23 mmol) in CH_2_Cl_2_ (0.30 mL) for 4 h at room temperature and the following bulb-to-bulb distillation (150–170 °C at 5 mmHg). Colorless crystals (CHCl_3_); Mp. 51.0–52.2 °C; IR (KBr) *ν* 2973, 2911, 2877, 1713, 1481, 1398, 1213, 1114, 1152, 677 cm^−1^; ^1^H-NMR (CDCl_3_) *δ* 1.22 (18H, s), 2.76 (2H, t, *J* = 7.1 Hz), 5.04 (1H, t, *J* = 7.1 Hz), 5.17 (1H, t, *J* = 7.1 Hz); ^13^C-NMR (CDCl_3_) *δ* 25.6, 27.3, 44.9, 52.3, 74.7, 119.2 (q, *J*_C-F_ = 329.8 Hz), 210.4; ^19^F-NMR (CDCl_3_) *δ* −10.0 (6F, s); MS (ESI-TOF) *m*/*z* 477 [M+H]^+^; HRMS calcd. for C_15_H_23_F_6_O_6_S_2_ [M+H]^+^, 477.0840; found, 477.0842.

*4-(2,2-Bis(trifluoromethylsulfonyl)ethyl)-2,6-dimethylheptane-3,5-dione* (**4i**). According to the general procedure, this compound was obtained in 80% yield (62.4 mg, 0.139 mmol) by the reaction of Tf_2_CHCH_2_CHTf_2_ (**5**, 100 mg, 0.175 mmol) with 2,6-dimethylheptane-3,5-dione (33 μL, 0.19 mmol) in CH_2_Cl_2_ (0.15 mL) for 2.5 h at room temperature and the following bulb-to-bulb distillation (150–165 °C at 5 mmHg). Colorless oil; IR (neat) *ν* 2979, 2941, 2880, 1725, 1469, 1396, 1213, 1114, 1024, 689 cm^−1^; ^1^H-NMR (CDCl_3_) *δ* 1.14 (6H, d, *J* = 6.7 Hz), 1.16 (6H, d, *J* = 7.0 Hz), 2.69–2.82 (4H, m), 4.73 (1H, t, *J* = 7.3 Hz), 5.32 (1H, t, *J* = 6.9 Hz); ^13^C-NMR (CDCl_3_) *δ* 17.8, 18.4, 24.5, 41.5, 57.3, 74.7, 119.2 (q, *J*_C-F_ = 329.7 Hz), 208.1; ^19^F-NMR (CDCl_3_) *δ* −10.3 (6F, s); MS (ESI-TOF) *m*/*z* 449 [M+H]^+^; HRMS calcd. for C_13_H_19_F_6_O_6_S_2_ [M+H]^+^, 449.0527; found, 449.0508.

*Triethyl 3,3-bis(trifluoromethylsulfonyl)propane-1,1,1-tricarboxylate* (**4j**). According to the general procedure, this compound was obtained in 88% yield (183 mg, 0.349 mmol) by the reaction of triethyl methanetricarboxylate (92.2 mg, 0.397 mmol) with Tf_2_CHCH_2_CHTf_2_ (**5**, 295 mg, 0.515 mmol) in acetonitrile (0.40 mL) at 80 °C for 13 h and the following removal of Tf_2_CH_2_ using Kugelrohr oven (100 °C at 5 mmHg). Colorless oil; IR (neat) *ν* 2988, 2943, 2911, 1745, 1393, 1220, 1110, 1018, 860, 701 cm^−1^; ^1^H-NMR (CDCl_3_) *δ* 1.28 (9H, t, *J* = 7.2 Hz), 3.45 (2H, d, *J* = 5.0 Hz), 4.27 (6H, q, *J* = 7.2 Hz), 6.53 (1H, t, *J* = 5.0 Hz); ^13^C-NMR (CDCl_3_) *δ* 13.6, 27.3, 62.1, 63.5, 73.9, 119.3 (q, *J*_C-F_ = 330.2 Hz), 165.4; ^19^F-NMR (CDCl_3_) *δ* −8.5 (6F, s); MS (ESI-TOF) *m*/*z* 525 [M+H]^+^; HRMS calcd. for C_14_H_19_F_6_O_10_S_2_ [M+H]^+^, 525.0324; found, 525.0299.

## 4. Conclusions

In summary, we successfully found that δ-oxo-1,1-bis(triflyl)alkanes are obtained in good to excellent yields by the reaction of enolizable carbonyls with Tf_2_CHCH_2_CHTf_2_ (**5**). NMR study of a solution of tetrasulfone **5** in CDCl_3_ revealed smooth formation of reactive 1,1-bis(triflyl)ethylene (**3**) in a reversible manner. On the basis of this reaction, incorporation of Tf_2_CH functionality into a wide range of 1,3-dicarbonyl compounds was realized. Furthermore, gas-phase acidities of some δ-oxo-1,1-bis(triflyl)alkanes thus obtained were determined by the FT-ICR technique. The present work is a notable extension of our synthetic methodology for Tf_2_CH type carbon acids. Further studies on this reaction and catalysis of the δ-oxo-1,1-bis(triflyl)alkanes are under progress in our laboratory.
